# Movement-Modulation of Local Power and Phase Amplitude Coupling in Bilateral Globus Pallidus Interna in Parkinson Disease

**DOI:** 10.3389/fnhum.2018.00270

**Published:** 2018-07-09

**Authors:** Nicholas AuYong, Mahsa Malekmohammadi, Joni Ricks-Oddie, Nader Pouratian

**Affiliations:** ^1^Department of Neurosurgery, University of California, Los Angeles, Los Angeles, CA, United States; ^2^Institute for Digital Research and Education, University of California, Los Angeles, Los Angeles, CA, United States; ^3^Neuroscience Interdepartmental Program, University of California, Los Angeles, Los Angeles, CA, United States; ^4^Brain Research Institute, University of California, Los Angeles, Los Angeles, CA, United States

**Keywords:** Parkinson disease, β oscillations, phase amplitude coupling, interhemispheric coordination, globus pallidus interna

## Abstract

There is converging evidence that bilateral basal ganglia motor networks jointly support normal movement behaviors including unilateral movements. The extent and manner in which these networks interact during lateralized movement remains unclear. In this study, simultaneously recorded bilateral Globus Pallidus interna (GPi) local field potentials (LFP) were examined from 19 subjects with idiopathic Parkinson disease (PD), while undergoing awake deep brain stimulation (DBS) implantation. Recordings were carried out during two behavioral states; rest and cued left hand movement (finger tapping). The state-dependent effects on α- β oscillatory power and β phase-encoded phase amplitude coupling (PAC), including symmetrical and assymetrical changes between hemispheres, were identified. Unilateral hand movement resulted in symmetrical oscillatory power suppression within bilateral GPi at α (8–12 Hz) and high β (21–35 Hz) and increase in power of high frequency oscillations (HFO, 200–300 Hz) frequency bands. Asymmetrical attenuation was also observed at both low β (13–20 Hz) and low γ (40–80 Hz) bands within the contralateral GPi (*P* = 0.009). In addition, unilateral movement effects on PAC were confined to the contralateral GPi with attenuation of both low β-low γ and β-HFO PAC (*P* < 0.05). Further analysis showed that the lateralized attenuation of low β and low γ power did not correlate with low β-low γ PAC changes. The overall coherence between bilateral GPi was not significantly altered with unilateral movement, however the preferred phase difference in the high β range increased from 0.23 (±1.31) radians during rest to 1.99 (±0.78) radians during movement execution. Together, the present results suggest that unilateral motor control involves bilateral basal ganglia networks with movement features differentially encoded by distinct frequency bands. The lateralization of low β and low γ attenuation with movement suggests that these frequency bands are specific to the motor act whereas symmetrical expression of α, high β, and HFO oscillations best correspond to motor state. The restriction of movement-related PAC modulation to the contralateral GPi indicates that cross-frequency interactions appear to be associated with lateralized movements. Despite no significant movement-related changes in the interhemispheric coherence, the increase in phase difference suggests that the communication between bilateral GPi is altered with unilateral movement.

## Introduction

The execution of daily manual tasks involves the coordination of bihemispheric cortical and subcortical motor areas (Cardoso de Oliveira et al., 2001; Gross et al., 2005; Pollok et al., 2005), including the basal ganglia (Wannier et al., 2002; Kraft et al., 2007). While lateralized (i.e., unilateral) manual movements are routine motor behaviors, the extent and manner in which bilateral motor networks contribute to movement lateralization is unclear. In general, movement lateralization is theorized to be shaped through a cortical interhemispheric inhibition mechanism, whereby contralateral hemisphere motor output is restricted via transcallosal pathways (Leocani et al., 2000; Liepert et al., 2001; Duque et al., 2005). The basal ganglia likely plays a central role in this process through indirect globus pallidus interna (GPi; Scholz et al., 2000; Lehéricy et al., 2006; Kraft et al., 2007) projections to the supplementary motor area (Muakkassa and Strick, 1979; Shibasaki and Hallett, 2006) via the thalamus (Hoover and Strick, 1993). Despite these observations, the precise neurophysiological mechanism for bihemispheric basal ganglia coordination during lateralized movement remains to be elucidated.

Oscillatory synchronization is posited as a key mechanism for functional integration across spatially distributed networks, including bihemispheric basal ganglia coordination (Baker et al., 1999; Buzsáki, 2004; Fries, 2005; Bonnefond et al., 2017), and may subserve movement lateralization. Supporting evidence stems from human studies on Parkinson disease (PD) subjects where bilateral subthalamic nuclei (STN) are engaged during unilateral hand movement (Alegre et al., 2005; Williams et al., 2005; Devos et al., 2006; Hebb et al., 2012; Darvas and Hebb, 2014; Kato et al., 2016). It is however difficult to interpret the functional significance of bilaterally coupled β oscillatory power within the STN in PD given that exaggerated subcortical oscillations is a hallmark neurophysiological feature of the disease in both human patients (Schnitzler and Gross, 2005; Brittain and Brown, 2014; Brittain et al., 2014; Little and Brown, 2014) and in corresponding animal models (Nini et al., 1995; Sharott et al., 2005; Stein and Bar-Gad, 2013; Dorval and Grill, 2014). Interestingly in PD where GPi activity is altered secondary to dopamine deficiency (Tremblay et al., 1989; Filion and Tremblay, 1991; Desikan et al., 2006; Wichmann and Soares, 2006; Moran et al., 2012; Devergnas et al., 2014), impairment of bimanual coordination (Johnson et al., 1998; Palmer et al., 2009) as well as movement lateralization (Cincotta et al., 2006; Wu et al., 2015) has been described.

Similarly, phase-amplitude coupling (PAC), where the phase of a lower-frequency oscillation modulates a higher frequency amplitude, has also been suggested to be a pathophysiological feature PD (de Hemptinne et al., 2013, 2015). While oscillatory power is largely an indicator of integrated neuronal synchronization from a localized area (Buzsáki and Watson, 2012), PAC likely reflects local or large scale inter-network communication subserved through cross-frequency interactions (Jensen and Colgin, 2007). Both cortical β-γ PAC (de Hemptinne et al., 2013, 2015; Swann et al., 2015; Kondylis et al., 2016) and STN β-HFO PAC have been shown to reflect PD severity (López-Azcárate et al., 2010; Yang et al., 2014; van Wijk et al., 2016). Moreover, therapeutic deep brain stimulation (DBS) at both STN and GPi has been shown to attenuate excessive cortical β-γ PAC (de Hemptinne et al., 2015; Malekmohammadi et al., 2018b) does dopaminergic therapy (Swann et al., 2015) similarly seen with levodopa induced attenuation of bilateral STN oscillations in the low β range (13–20 Hz; Little et al., 2013).

Despite the correspondence of both oscillatory power and PAC with PD symptoms, there is converging evidence that oscillatory power and PAC operate in a concerted fashion in bilateral basal ganglia motor networks to shape motor control (Kondylis et al., 2016). In healthy rodents, frequency-specific movement modulation of bilateral corticostriatal oscillatory power and PAC has been reported (von Nicolai et al., 2014). Similarly, Kato et al. (2016) showed that both local oscillatory power and PAC in bilateral STN of PD subjects exhibit frequency-specific modulation between rest and sustained voluntary wrist contraction. They found that while bilateral α/β oscillatory power and β-γ PAC are symmetrically attenuated in bilateral STN with movement, theta-gamma PAC emergence is asymmetrical and occurs only in the contralateral STN. The dissociation confirms (as one would suspect) that motor behaviors are driven through both bilaterally coupled and also hemisphere-specific neurophysiological changes of the basal ganglia.

A greater understanding of lateralized movement effects on bilateral GPi oscillations is important for several reasons: (1) in contrast to the STN with known cortico-STN bidirectional coupling (Lalo et al., 2008) through the cortical-STN hyperdirect pathway (Alexander and Crutcher, 1990; Parent and Hazrati, 1995; Nambu et al., 2002), there are no known direct cortico-GPi connections. As such, confirmation of concurrent bilateral and unilateral engagement of GPi during unilateral movement would indicate a generalizable principle of coordinated bihemispheric basal ganglia-thalamocortical (BGTC) network activity to support lateralized movements. (2) Identification of oscillatory signatures of movement lateralization will further clarify the role of bilateral basal ganglia in distributed motor control. (3) Neuromodulation therapeutic development for movement disorders, including closed-loop DBS system for PD, will benefit from identification of narrow-band channels associated with motor control. In this study, the extent and state dependency of bihemispheric GPi oscillatory patterns relevant to ipsilateral limb control, were examined in PD human subjects during two behavioral states; rest and unilateral hand movement. Narrow-band analysis of β oscillatory power and β phase-encoded PAC between bilateral GPi were carried out to identify symmetrical and asymmetrical movement-related changes.

## Materials and Methods

### Patients and Surgical Procedure

Nineteen subjects with idiopathic PD (15 males and 4 females) undergoing bilateral GPi DBS lead implantation, provided informed written consent in accordance with the Declaration of Helsinki and approved by the institutional review board at the University of California, Los Angeles, CA, USA (Study number: 16-000902). The average (± standard deviation) age was 63.5 (±5.8) years (Table 1). Clinical trials have established equal motor efficacy between DBS at both GPi and STN (Anderson et al., 2005; Deuschl et al., 2006). The decision to target GPi was based on clinical evidence of equal motoric efficacy, discussion with the patient of potential benefits and side effects, and the recommendations of an interdisciplinary clinical team that includes a neurosurgeon and movement-disorder neurologist. The indications for DBS therapy were based on those described in previous large scale randomized clinical trials (Weaver et al., 2009).

**Table 1:** Cohort demographics and relevant clinical information.

Subject	UPDRS-PIII^OFF,1^	UPDRS-PIII^ON,2^	Disease duration^3^	Levodopa equivalent dosage (mg)	More effect body side	Hoehn and yahr stage	DBS contacts used for chronic stimulation (6–10 months follow up) positive contact, negative contact, voltage (volts)/pulse-width (μs)/stimulation frequency (Hz)	
							Left lead	Right lead
S1	32	12	7	1420	L > R	2	C+, 2−, 4.0/90/160	C+, 11−, 4.4/90/160
S2	N/A	N/A	8	850	L > R	2	C+, 1−, 2.0/90/160	C+, 9−, 2.3/90/160
S3	35	18	9	1714	L > R	3	C+, 1−, 1.0/90/160	C+, 9−, 1.0/90/160
S4	51	25	18	848	R > L	3	C+, 1−, 1.5/90/160	C+, 9−, 1.0/90/160
^4^S5	31	15	5	1180	R > L	2	C+, 1−, 1.0/90/160	C+, 9−, 1.5/90/160
^4^S6	29	21	9	1000	L > R	2	C+, 1−, 1.5/90/160	C+, 9−, 1.7/90/160
^4^S7	27	9	7	1430	R > L	2	C+, 1−, 2.0/120/160	C+, 10−, 2.0/90/160
S8	54	20	17	1325	L > R	3	C+, 1−, 2.9/60/130	C+, 9−, 3.9/60/130
S9	42	17	21	1100	L > R	3	C+, 1−, 2.3/90/160	C+, 9−, 2.3/90/160
S10	37	11	12	915	R > L	2	C+, 1−, 3.0/90/185	C+, 9−, 30/90/185
S11	35	14	7	1800	L > R	2	C+, 2−, 3/90/160	C+, 9−, 4.0/90/160
S12	57	8	7	2483	L > R	4	C+, 2−, 3.2/90/180	C+, 9−, 4.4/90/180
S13	22	9	5	2412	L > R	3	C+, 1−, 1.0/90/160	C+, 9−, 1.0/90/160
S14	23	5	5	1475	L > R	1	C+, 1−, 2.8/90/160	C+, 9−, 4.0/90/160
S15	52	14	19	860	L > R	4	2+, 1−, 2.2/90/160	10+, 9−, 2.7/90/160
^4^S16	37	19	6	1175	R > L	2	C+, 1−, 2.0/90/180	C+, 10−, 2.0/90/180
^4^S17	N/A	10	12	935	L > R	3	C+, 1−, 1.5/90/160	C+, 9−, 1.5/90/160
S18	35	11	11	1250	L = R	2	C+, 2−, 3.1/90/130	C+, 8−, 2.7/60/130
S19	34	14	8	600	R > L	2	C+, 1−, 2.9/90/160	C+, 9−, 2.4/90/160

*^1^Examination was performed in the OFF-medication state. ^2^Examination was performed in the ON-medication state. ^3^Years since diagnosis. ^4^Initial programming parameters are reported, in the last column no later follow-up available*.

Bilateral GPi local field potentials (LFP) were recorded simultaneously during rest and cued left hand movement (finger tapping) while subjects were undergoing awake DBS implantation. All subjects underwent a detailed pre-operative neurological evaluation that included clinical grading using the Unified Parkinson Disease Rating Scale (UPDRS) motor (part III) both off and on medication. All patients underwent the surgical procedure in the off-medication state; all medications were withdrawn at least 12 h prior to the surgical procedure. All anesthetic medications were withheld for at least 1 h prior to recordings. Pre-operative clinical scores (i.e., UPDRS, Part 3, motor examination) was 39 (±13) and 14 (±5) when tested OFF and ON medication, respectively (Table 1).

All subjects underwent clinical pre- and post-operative imaging. Pre-operative imaging included T1-weighted magnetization prepared rapid acquisition gradient echo (MPRAGE) image (slice thickness = 1 mm, repetition time = 2100 ms, echo time, 2.98 ms, flip angle = 15°, 3T, Siemens Skyra). For implantation, a Leksell stereotactic headframe (Elekta Instruments, Stockholm, Sweden) was applied to the skull and a full head computed tomography (CT) scan was obtained using 0.6 mm slice thickness (Siemens Sensation 64). The DBS lead (Model 3387, 1.27 mm lead body diameter, contact length 1.5 mm, inter-contact distance 1.5 mm, Medtronic Inc., Minneapolis, MN, USA) was targeted to motor (ventral posterolateral) GPi using image-guided targeting, 2–4 mm anterior, 19–24 mm lateral and 4–6 mm inferior to the mid-commissural point (depending on individual anatomy). All trajectories were confirmed with intraoperative microelectrode recordings based on firing activity and awake macrostimulation testing. Details of surgical technique, including targeting, are provided in prior publications (Tsiokos et al., 2013, 2017).

### Data Recording and Pre-processing

After completion of the clinical testing, research-related recordings were performed. LFPs were recorded with the lead in final implant position in all subjects using the lead’s four ring electrode contacts (contacts 0–3 oriented ventral to dorsal respectively). Signal acquisition was performed using BCI2000 v3 connected to an amplifier (g.Tec, g.USBamp 2.0) using a sampling rate of 2400 Hz and online 0.1–1000 Hz band-pass filtering. All patients were right-handed and wore a sensor glove (5DT data glove 5 Ultra) on the left hand to provide concurrent hand movement recordings at a slower effective sampling rate which was oversampled offline at 2400 Hz by BCI2000 using stair step interpolation. Ground and reference contacts were connected to the scalp. Bipolar re-referencing was used for further signal analysis, yielding two bipolar signals to evaluate signals recorded from GPi (bipolar pair 0–1). This contact pair was used across the cohort and conditions to ensure anatomic consistency across subjects (Tsiokos et al., 2017), ultimately corresponding to the site of chronic stimulation in most patients (Table 1). While not utilized in this analysis, a cortical electrocorticography (ECoG) strip was introduced subdurally in a posterior direction through the implant burr hole for other experiments. Because the clinical protocol of the institution is to implant the left and then the right sided lead when performing bilateral implants, this necessitated evaluation of left hand movements as the ECoG strip was necessarily placed through the right sided (second) burr hole.

Each subject performed a block-design finger tapping task alternating between 30 s blocks of rest and left-hand cued finger tapping. Subjects were given verbal cues. They were instructed to remain as still as possible while keeping their eyes open during the resting condition. Movement was initiated following a verbal cue at which point the subjects started to open and close their left hand at their maximum amplitude and fastest comfortable speed. The quality of the movement was monitored visually by a member of experimental team as well as recorded finger joints data from the sensor glove. Up to six trials were recorded from each subject. Some trials were also omitted due to the unwanted movement or electrical noise during recordings. We therefore used the first two noise-free rest-movement trials from each subject in this study.

Signal analysis was performed using custom made scripts in MATLAB (Version 8.6, The Mathworks Inc., Natick, MA, USA) and Fieldtrip toolbox for EEG/MEG-analysis (Oostenveld et al., 2011). Data was separated into rest and movement epochs excluding all the segments containing electrical or unwanted movement artifact. Raw ECoG and LFPs were first subjected to automatic artifact rejections and verified through visual inspection with an interactive waveform browser, capable of user selection of artifact segments. Automatic artifact detection was accomplished by first performing a full-wave rectification, then taking the first derivative, and subsequently filtering with a five-point median filter to identify waveform segments that exceeded five standard deviations of the entire recorded session for each subject. Location windows of artifact segments were extended by 2 ms on each side. Each artifact segments were then replaced with a linear interpolation based on surrounding data. Removed segments primarily featured power spectra with abnormally high values, excessive harmonics and time series with high rates of voltage change. Data were bandpass filtered from 1 Hz to 300 Hz using a two-way least square FIR filtering (eegfilt.m, forward and backward to ensure no phase distortion was created during band pass filtering) 60 Hz line-noise and its harmonics (up to 300 Hz) were removed from the data using a notch filter implemented in the fieldtrip toolbox.

### Power Spectral Density (PSD)

Power spectral density (PSD) for “rest” and “movement” conditions were calculated using the Thomson’s multitaper method in 1 s consecutive time windows with no overlap for frequencies of 1–300 Hz with ±2 Hz frequency bandwidth (three taper; Bokil et al., 2010). Group average PSDs for both conditions were then calculated for the left and right GPi signals separately. To account for inter-subject variability in baseline power, each segmented spectrum was normalized to the total power of the signal during each condition (excluding the line-noise and its harmonics).

### Coherence and Imaginary Coherence

To describe the degree of co-variability, we estimated magnitude squared coherence between left and right GPi signals. Magnitude squared coherence was calculated using multitaper method with time window and frequency smoothing parameters identical to the analysis of PSD (frequencies from 1 Hz to 300 Hz with ±2 Hz frequency bandwidth and three tapers).

We further explored effects of spurious volume conduction using imaginary coherence (iCoh; Nolte et al., 2004) which has been shown to be insensitive to volume conduction of common signals. Using similar parameters as that used for coherence analyses, we derived the imaginary part of coherency between the two signals for non-overlapping time windows. To assess statistical significance of differences in group average iCoh between two conditions (i.e., rest and movement), we used permutation testing, pooling together values from all time segments for all subjects (considering that tapers are orthogonal and time windows are non-overlapping, we could reasonably assume that tapered Fourier transforms are interchangeable). At each permutation (*n* = 10,000), a random subset of the cohort was selected for which the labels of the conditions were swapped allowing to create a null distribution of the group difference. Since the difference values at each frequency are bound between −1 and 1, the Fisher Z-transform of the condition difference could be assumed approximately normally distributed under the null hypothesis. Significance of the condition difference was then assessed using *P* = 0.05 and corrected for multiple comparisons in a similar fashion to spectral and coherence statistical analysis.

The “preferred phase difference” of coupling for each frequency value was also calculated as the circular average (Berens, 2009) of the phase difference values across all time windows for each condition separately. The avergae of iCoh values and the preferred phase difference were calculated for low and high β frequency bands for each subject and used for further statistical comparison between rest and movement conditions.

### Phase Amplitude Coupling (PAC)

Phase amplitude coupling (PAC) was estimated using Tort’s method of Modulation Index (MI; Tort et al., 2010; Malekmohammadi et al., 2015). Parameters selected for PAC analysis were chosen using based on recommendations from a previous critical analyses of PAC methodology (Aru et al., 2015). Briefly signals were band pass filtered using a two-way least squares FIR filtering (phase: 1–35 Hz, in 1 Hz steps and 2 Hz bandwidth; amplitude: 1–300 Hz in 2 Hz steps and double the phase-encoding frequency). Hilbert transform was then used to extract instantaneous phase and amplitude of the two components, respectively. Phase values were then binned (18 bins, 20° width) and mean amplitude distribution was calculated relative to the phase bins to create a phase-amplitude histogram. Kullback-Leibler (KL) divergence was used to measure deviation of this histogram from uniform distribution and create MI values. In order to extract the preferred phase, the weights of the phase-amplitude histogram were used as amplitudes of a vector, while the center phase of each bin constituted the phase of the vector.

### Statistical Analysis

Statistical analysis was performed using Matlab, SPSS statistical software (IBM Corp.) and STATA (StataCorp LLC). Statistically significant differences in spectral power/coherence between two conditions (rest vs. movement) at each frequency (1–300 Hz) was assessed using the two-group test of the spectrum/coherence (Bokil et al., 2007), with a null hypothesis that both conditions have equal spectra/coherence within the cohort. The statistical properties of asymptotic distributions for power spectra and magnitude squared coherence are well known (Bokil et al., 2007; Malekmohammadi et al., 2018a). Our choice of the two-group test was based on the asymptotic probability distributions and jackknife correction of the difference z-scores. This method is advantageous for its correction of bias inherent to the estimation process (Malekmohammadi et al., 2018a). Since multitaper analysis uses an orthogonal family of tapers (i.e., Slepian sequences) calculated using non-overlapping windows, the calculated tapered spectra/coherence can be reasonably assumed to be statistically independent. We derived the mean group spectra/coherence for both conditions along with the corresponding Z statistics using asymptotic probability distribution. The 95% confidence intervals were then calculated based on the Jackknife estimation of variance as previously described (Bokil et al., 2007). To address the issue of multiple comparisons, we note that differences in spectra/coherence due to chance are likely to be at discrete frequencies, while neurophysiological differences spans contiguous frequency ranges (i.e., α, β). Since spectral/coherence estimates at frequencies separated by less than the bandwidth of the multitaper method (4 Hz) are inherently correlated, we rejected the null hypothesis for all candidate frequencies constituting bands whose width is larger than 4 Hz (Goldfine et al., 2011).

To assess the statistical significance of the PAC values, surrogate data analysis using a shuffling procedure was performed. For each signal pair, we generated 1000 temporally shuffled versions of phase signals and calculated MI values for each. The true MI value was converted to a Z-score and the false discovery rate (FDR) procedure was used with *q* = 0.05 to adjust the corresponding *p-values* and correct for multiple comparisons (Benjamini et al., 2001; Genovese et al., 2002). For each frequency pair with statistically significant PAC, the “preferred phase of coupling” was found as the phase bin with maximal amplitude measurement. The circular mean of the preferred phase was calculated for all the frequency pairs in distinct frequency bands for each patient. Average PAC was calculated for distinct frequency band pairs and used for further statistical analyses across the population. Finally phase and amplitude frequencies involved in maximal PAC were examined across the cohort.

In addition to the frequency-by-frequency statistical analysis introduced above, the average band power values were calculated for the different frequency bands (α: 8–12 Hz), low β (13–20 Hz), high β (20–35 Hz), low γ (40–80 Hz) and HFO (200–300 Hz). The Shapiro-Wilk test was used to assess normality of distribution prior to comparing power (at different frequency bands) and PAC. This test was chosen as the sample size of this study is smaller than 50. If normality condition was not satisfied, non-parametric paired sample Wilcoxon signed-rank test and otherwise paired sample *t*-test was selected to compare power and PAC across pallidal contacts. All resulting *p-values* were corrected for multiple comparisons using Bonferroni or Holm’s sequential Bonferroni method (Holm, 1979).

## Results

### Unilateral Hand Movement Modulates Bilateral GPi Oscillatory Power

**Figure 1:**
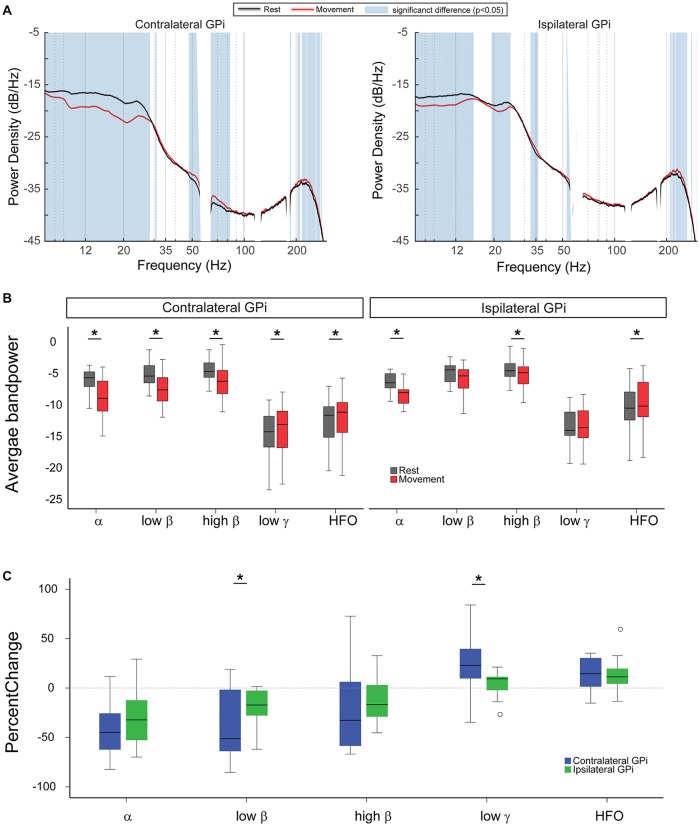
Unilateral hand movement modulates oscillatory power in bilateral Globus Pallidus interna (GPi). **(A)** Average power spectral densities across the cohort during rest (black curve) and unilateral finger tapping (red curve). Blue vertical shade indicates significant difference between resting and movement conditions as tested by two-group test of spectra and corrected for multiple comparisons. **(B)** Comparing average power for different frequency bands between rest (gray) and movement (red). **(C)** Comparison of percent change in the spectral power between the two GPi ipsilateral (green) and contralateral (blue) to the moving body side ((Power during movement − Power during rest)/Power during rest ×100). In panels **(B,C)**, asterisk signs (*) indicate statistical significance of the difference between the two conditions as tested by paired *t*-test and corrected for multiple comparisons (*P* < 0.05).

During rest, oscillatory power was symmetrically expressed in bilateral GPi, with greatest power at the α and β frequency ranges. With unilateral movement, power modulation within bilateral GPi occurred over a wide frequency range. Using the two-group test for spectral power, we observed that movement induced power suppression for frequencies between 8 Hz and 29 Hz (α and β range) in the contralateral GPi concomitantly with an increase in power at frequencies between 48 Hz and 83 Hz (excluding the line noise 58–62 Hz, low γ range) and 204–274 Hz (HFO range). At the ipsilateral GPi, power in the low frequencies (8–15 Hz) and (19–25 Hz) was similarly suppressed with movement while power at high frequencies (210–262 Hz, HFO) was significantly increased (Figure 1A).

Analysis of movement-related changes in average band power revealed similar results, while also enabling further comparison of relative changes across the hemispheres (Figures 1B,C). Unilateral hand movement similarly suppressed α and high β power and increased HFO power in ipsi- and contralateral GPi. Specifically, left-hand movement significantly suppressed average α and high β power in contralateral GPi (−35.52 (±8.99)%, *p* = 0.002 and −23.8 (±9.81)%, *p* = 0.009, respectively, paired *t*-test) as well as the ipsilateral GPi both in the ipsilateral (−21.54 (±12.05)%, *p* = 0.016 and −12.35 (±4.81)%, *p* = 0.03, respectively, paired *t*-test, Figure 1B). The magnitude of power suppression did not differ significantly across hemispheres for these two bands (*p* = 0.18 and 0.4, respectively, paired *t*-test, Figure 1C). Likewise, movement related increase of the HFO power was similar between contralateral (13.57 (±3.83)%, *P* = 0.009, paired *t-test*) and ipsilateral GPi (15.72 (±4.79)%, *P* = 0.009, paired *t-test*).

In contrast, unilateral hand movement differentially modulated low β and low γ GPi power between the hemispheres. Significant changes in low β and low γ GPi power were only observed within contralateral GPi (−28.08 (±12.83)%, *p* = 0.009 and 22.99 (±5.87)%, *p* = 0.009, respectively, paired *t*-test, Figure 1B). Movement related power changes in the ipsilateral GPi were not significant (low β: −10.62 (±8.99)%, *p* = 0.15 and low γ: 3.84 (±2.66)%, *p* = 0.71). Accordingly, the percent power change for low β and low γ was significantly greater in the contralateral GPi (*p* = 0.002 and 0.036, respectively, paired *t*-test, Figure 1C).

### Movement Modulates Contralateral GPi Low β-Low γ PAC

**Figure 2:**
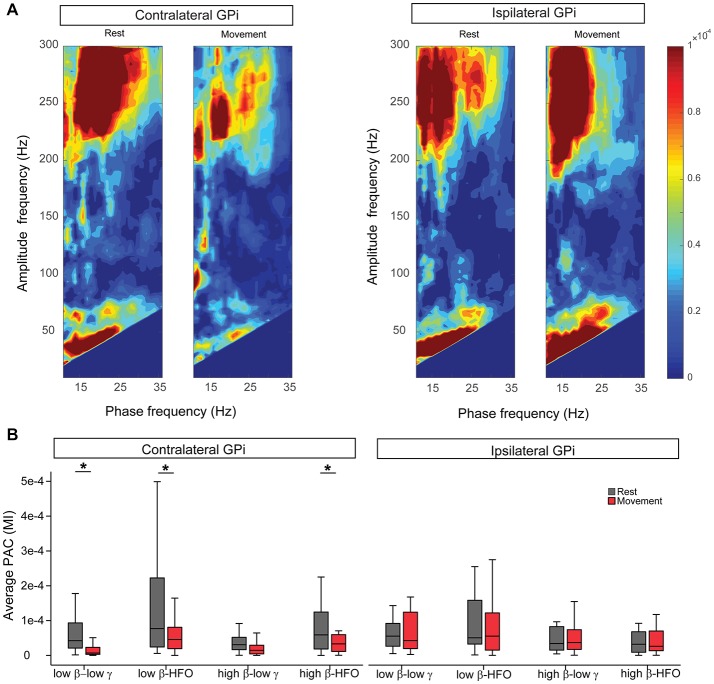
Unilateral hand movement modulates phase-amplitude coupling (PAC) only at the GPi contralateral to the moving body side. **(A)** Average comodulogram (MI values) for the cohort at bilateral GPi during rest and movement conditions. **(B)** Comparison of average PAC values at different frequency bands during rest (gray) and movement (red) indicates significant suppression of the PAC only at the GPi contralateral to the movement side. Asterisk signs (*) indicate statistical significance of the difference between the two conditions as tested by Wilcoxon signed-rank test and corrected for multiple comparisons (*P* < 0.05). Each box contains the interquartile range and whiskers extend to the highest and lowest observations.

At rest, β-low γ and β-HFO PAC were similarly expressed in bilateral GPi, for both low and high β phase encoding frequencies (Figure 2A). At rest, the β-low γ PAC was maximal at a phase-encoding frequency of 17.68 (±1.25) Hz in the contralateral GPi and 17.37 (±0.77) Hz for the ipsilateral GPi (no difference between hemispheres, *p* = 0.77, Wilcoxon signed-rank test). Analysis of preferred phase for resting low β-low γ PAC revealed an average preferred phase for coupling of 0.23 (±0.07) radians in the contralateral GPi and 0.28 (±0.05) in the ipsilateral GPi (no difference between hemispheres, *p* = 0.45, Wilcoxon signed-rank test). With unilateral hand movement, contralateral GPi low β-low γ PAC was significantly suppressed (−45.67 (±16.23)%, *p* = 0.004, Wilcoxon signed-rank test, Figure 2B). Although contralateral low β-low γ PAC suppressed with movement, the maximal phase-encoding frequency in the contralateral GPi did not change with movement (*P* = 0.24, Wilcoxon signed-rank test). Likewise, unilateral movement resulted in no significant change in the preferred phase of coupling at the contralateral GPi (*P* = 0.75, Wilcoxon signed-rank test). Movement-related changes in low β-HFO and high β-HFO coupling were also significant within the contralateral GPi (*P* = 0.01 and 0.03 respectively Wilcoxon signed-rank test) while high β-low γ PAC was not significantly modulated by contralateral hand movement. Notably, despite similar resting PAC profiles between contralateral and ipsilateral GPi, unilateral hand movement did not result in any significant changes in ipsilateral GPi PAC (Figure 2).

To delineate the potential effect of movement related power changes (low and high β, low γ and HFO) on PAC suppression, we investigated whether those changes correlated with PAC changes. Such analysis showed that only high β-HFO PAC changes were correlated with changes in high β power (Pearson’s correlation *R* = 0.62, *P* = 0.016, corrected for multiple comparisons). No other significant correlation was found between low β or low γ power changes and low β-low γ PAC changes. Likewise, there was no significant correlation between low β or HFO power changes and low β-HFO coupling. HFO power changes and high β-HFO PAC changes were not significantly correlated. Finally, none of the PAC changes in the ipsilateral GPi were significant.

### Bilateral GPi Are Coherent at High β Frequencies Regardless of Movement Condition

**Figure 3:**
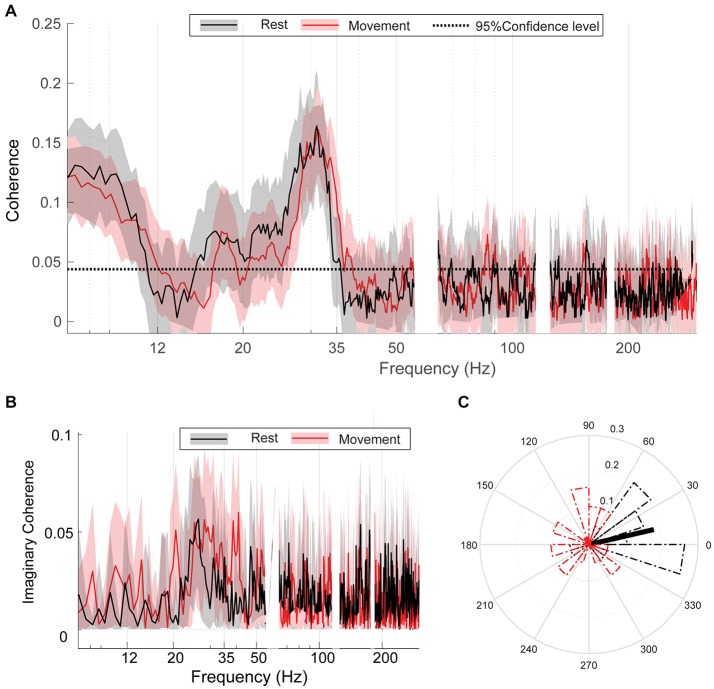
Bilateral GPi are coherent regardless of movement condition. **(A)** Group average coherence between bilateral GPi during rest (black) and movement (red) indicating statically significant coherence (greater that 95% confidence level: dashed black line) which does not differ between conditions. **(B)** Group average imaginary coherence (iCoh) during rest (black) and movement (red). **(C)** Circular histograms of the preferred phase difference between bilateral GPi during rest (black) and movement (red). Thick black/red lines indicate direction of mean phase difference between the two nuclei during rest/movement. Each box contains the interquartile range and whiskers extend to the highest and lowest observations.

At rest, bilateral GPi exhibited significant coherence in both α (8–11 Hz) and β (15–35 Hz) frequency ranges (Figure 3A). Unilateral hand movement did not significantly change the coherence spectrum between bilateral GPi (Figure 3A). The peak coherence frequency was located at 31.05 (±2.31) and 31.64 (±3.86) Hz during rest and movement, respectively, with no significant change between the two conditions (*p* = 0.35, Wilcoxon signed-rank test). Despite no change in overall coherence, the preferred phase difference between bilateral GPi in high β range changed significantly from 0.23 (±1.31) radians during rest to 1.99 (±0.78) radians with movement (*p* = 0.028, Kuiper’s test, Figure 3C). Like coherence, iCoh likewise did not change with movement (permutation testing, Figure 3B). Of note, the α peak noted in the coherence spectrum was absent in the iCoh spectrum.

## Discussion

Our results demonstrate that unilateral movement is associated with both symmetrical and asymmetrical modulation of bilateral GPi activity in a frequency-specific manner. Symmetrical modulation of oscillatory power including bilateral attenuation of α and high β power was observed along with bilateral HFO enhancement. In contrast, movement-modulation of low β and low γ power as well as low β-low γ PAC was asymmetric and only modulated within the contralateral GPi. Together, these findings suggest that bilateral BGTC motor networks support unilateral movement behaviors through a mechanism that includes interhemispheric coupling of basal ganglia oscillations (particularly in the α and high β) frequencies in conjunction with lateralized frequency-specific and cross-frequency interactions. The network-wide importance of α and high β is highlighted by their symmetric power suppression yet unchanged coherence with unilateral movement. Conversely, these results demonstrate that low β and low γ dynamics is specific for movement laterality. Although movement attenuation of low β power, low γ power and low β-low γ PAC was confined to the contralateral GPi, power changes was not correlated with PAC changes. This suggests that these processes are not linearly related and possibly distinct.

The mechanism underlying bihemispheric GPi coupling is not obvious given no known palido-pallidal interhemispheric pathways. As such, bi-hemispheric coupling likely occurs as a part of a more expansive bi-hemispheric network spanning cortical and subcortical areas. Indeed, sensorimotor cortical α and β oscillations have also been shown to be inter-hemispherically coupled in normal human subjects, as suggested by their bi-hemispheric desynchronization during unilateral movement (Chatrian et al., 1959; Crone et al., 1998). For example, electroencephalogram (EEG) studies in normal human subjects have long shown that both sensorimotor cortical α (also referred to as mu rhythms (Niedermeyer, 1997)) and β oscillations (Gastaut, 1952) predominate during rest but desynchronize prior to movement activity (Chatrian et al., 1959; Niedermeyer, 1997; de Solages et al., 2010; Avanzini et al., 2012; Stein and Bar-Gad, 2013; Brittain and Brown, 2014). Likewise, movement-related β and γ modulation occurs in bilateral STN during unilateral hand movement also in PD patients (Alegre et al., 2005; Kato et al., 2016). Our present result suggests that movement-modulation of bi-hemispheric cortical and subcortical oscillations is a feature of a generalized coupled bi-hemispheric BGTC network.

Interestingly, exaggerated α and β oscillations have been described in bilateral sensorimotor cortices in early-state PD along with a dampened desynchronization during movement (Pollok et al., 2012). Similarly, bilateral STN in PD also exhibit exaggerated β oscillations (Bronte-Stewart et al., 2009) along with an exaggerated β interhemispheric STN-STN (de Solages et al., 2010) and cortico-STN coupling (Kato et al., 2015). While it is not possible to differentiate the physiological and pathophysiological aspects of α and high β frequencies in this study, one conclusion is that their dynamics are most consistent with neural correlates of global motor state rather than specific movement feature, given the non-conformity to laterality of movement.

In contrast, low β and low γ oscillatory power are movement-modulated only in contralateral GPi. The dissociation between low and high β with movement suggests that high and low β frequencies likely have distinct functional roles. There are several pieces of evidence that suggests that low β coupling subserves normal motor control operations while high β coupling is likely pathophysiological (van Wijk et al., 2017; Malekmohammadi et al., 2018b). Hemiparkinsonian animal studies have shown that while low β power is reduced with motor activity, high β activity increases with movement at the motor cortex and basal ganglia in the dopamine-depleted hemisphere and is also significantly reduced with L-Dopa administration (Avila et al., 2010; Brazhnik et al., 2014; Delaville et al., 2014). Likewise, we have recently shown that therapeutic GPi-DBS only modulates high β motor cortical PAC without significant modulation of local β oscillatory power. In contrast, cortical oscillatory power in low β is modulated by contralateral hand movement, suggesting that it is most likely encoding the normal movement (Malekmohammadi et al., 2018b). Our present results are in agreement with these findings and further suggests that whereas high β oscillatory power and network coherence is an indicator of global motor state, low-β encoded PAC is consistent with a movement-specific channel.

The bilateral coupling and modulation of STN (Kato et al., 2016) and GPi activity during unilateral movement behaviors indicate that lateralized movement still involves bihemispheric BGTC network coordination. Generally, movement lateralization is theorized to be shaped through a cortical interhemispheric inhibition mechanism, whereby the motor network in the hemisphere ipsilateral to movement is inhibited by the contralateral hemisphere presumably via a transcallosal pathway (Leocani et al., 2000; Liepert et al., 2001; Duque et al., 2005). The supplementary motor area has been proposed to be a key cortical region involved in this process (Muakkassa and Strick, 1979; Shibasaki and Hallett, 2006). In addition, previous functional imaging studies in healthy subjects found that unilateral task performance normally involves bilateral activation of the sensorimotor putamen and GPi (Scholz et al., 2000; Lehéricy et al., 2006; Kraft et al., 2007). The GPi, a major output basal ganglia nuclei, indirectly projects to the SMA via the thalamus (Hoover and Strick, 1993) and therefore may play an important role in this process. Interestingly in PD, where GPi activity is altered secondary to dopamine deficiency (Tremblay et al., 1989; Filion and Tremblay, 1991; Desikan et al., 2006; Wichmann and Soares, 2006; Moran et al., 2012; Devergnas et al., 2014), there is impairment of bimanual coordination (Johnson et al., 1998; Palmer et al., 2009) as well as movement lateralization (Cincotta et al., 2006; Wu et al., 2015). Since there are no known direct interhemispheric connections between the basal ganglia (Beaulé et al., 2012), the underlying process of bihemispheric basal ganglia coordination likely relies on indirect network communications. Based on our present findings, we suggest that movement lateralization involves interhemispheric modulation which is permissive of movement, whereas specific movement features are encoded via lateralized modulation of frequency-specific power and cross-frequency interactions (Kato et al., 2016). While the degree of coherence between bilateral GPi was unaltered with movement, the preferred phase difference between bilateral GPi in high β range increased significantly. The maintained coherence between rest and movement may reflect state-independent communication taking place between bilateral GPi neuronal networks (Fries, 2005; Womelsdorf et al., 2007). The observed relative phase shift may represent a switch in the communication mode or information streams between bilateral GPi with unilateral movement (Tiesinga and Sejnowski, 2010; Maris et al., 2016). Interestingly, in a similar analysis of interhemispheric pallidal coherence in patients with dystonia, high beta coherence was not noted, providing further evidence of the pathophysiologic nature of network-wide high beta coherence (Neumann et al., 2015).

As this study was necessarily only completed in subjects undergoing DBS implantation surgery, we are limited in our ability to generalize these results to normal healthy humans. However, bihemispheric modulation of EEG signals with movement in healthy subjects is well documented phenomena (Gastaut, 1952; Niedermeyer, 1997). Furthermore, similar movement modulation of oscillatory power and PAC are also observed in healthy animals (von Nicolai et al., 2014) and correspond well with findings in hemiparkisonian animals (Avila et al., 2010; Brazhnik et al., 2014; Delaville et al., 2014). The present results largely agree with previous studies of the STN in PD subjects (Alegre et al., 2010; Kato et al., 2016) with some differences. Kato et al. showed that theta-gamma PAC was enhanced in the contralateral STN to sustained motor contraction where as our present study found low β-low γ PAC attenuation, along with enhancement in low β-HFO and high β-HFO PAC at the contralateral GPi to finger tapping. These dissimilarities may be due to the different motor tasks employed (i.e., sustained action vs. repetitive movement), the different nuclei studies (i.e., STN vs. GPi), or both. Despite inherent limitations, the symmetrical modulation of frequency-specific oscillatory power and asymmetrical modulation of the power of other frequency bands and PAC with unilateral movement, lend support to the concept of neural oscillations as well as higher-order oscillatory interactions subserves distinct normal sensorimotor functions (Sanes and Donoghue, 1993; Murthy and Fetz, 1996).

## Author Contributions

NA, MM and NP designed the research and performed the experiments. NP performed the surgery and treated patients, pre and post operation. NA and MM performed the data analysis. JR-O oversaw the statistical analyses and design. All authors participated in the manuscript writing.

## Conflict of Interest Statement

The authors declare that the research was conducted in the absence of any commercial or financial relationships that could be construed as a potential conflict of interest.

## References

[B1] Alegre M. Alonso-Frech F. Rodríguez-Oroz M. C. Guridi J. Zamarbide I. Valencia M.. (2005). Movement-related changes in oscillatory activity in the human subthalamic nucleus: ipsilateral vs. contralateral movements. Eur. J. Neurosci. 22, 2315–2324. 10.1111/j.1460-9568.2005.04409.x 16262669

[B2] Alegre M. Rodríguez-Oroz M. C. Valencia M. Pérez-Alcázar M. Guridi J. Iriarte J.. (2010). Changes in subthalamic activity during movement observation in Parkinson’s disease: is the mirror system mirrored in the basal ganglia? Clin. Neurophysiol. 121, 414–425. 10.1016/j.clinph.2009.11.013 20006544

[B3] Alexander G. E. Crutcher M. D. (1990). Functional architecture of basal ganglia circuits—neural substrates of parallel processing. Trends Neurosci. 13, 266–271. 10.1016/0166-2236(90)90107-l 1695401

[B4] Anderson V. C. Burchiel K. J. Hogarth P. Favre J. Hammerstad J. P. (2005). Pallidal vs. subthalamic nucleus deep brain stimulation in Parkinson disease. Arch. Neurol. 62, 554–560. 10.1001/archneur.62.4.554 15824252

[B5] Aru J. J. Aru J. J. Priesemann V. Wibral M. Lana L. Pipa G.. (2015). Untangling cross-frequency coupling in neuroscience. Curr. Opin. Neurobiol. 31, 51–61. 10.1016/j.conb.2014.08.002 25212583

[B6] Avanzini P. Fabbri-Destro M. Dalla Volta R. Daprati E. Rizzolatti G. Cantalupo G. (2012). The dynamics of sensorimotor cortical oscillations during the observation of hand movements: an EEG study. PLoS One 7:e37534. 10.1371/journal.pone.0037534 22624046 PMC3356327

[B7] Avila I. Parr-Brownlie L. C. Brazhnik E. Castañeda E. Bergstrom D. A. Walters J. R. (2010). β frequency synchronization in basal ganglia output during rest and walk in a hemiparkinsonian rat. Exp. Neurol. 221, 307–319. 10.1016/j.expneurol.2009.11.016 19948166 PMC3384738

[B8] Baker S. N. Kilner J. M. Pinches E. M. Lemon R. N. (1999). The role of synchrony and oscillations in the motor output. Exp. Brain Res. 128, 109–117. 10.1007/s002210050825 10473748

[B9] Beaulé V. Tremblay S. Théoret H. (2012). Interhemispheric control of unilateral movement. Neural Plast. 2012:627816. 10.1155/2012/627816 23304559 PMC3523159

[B10] Benjamini Y. Drai D. Elmer G. Kafkafi N. Golani I. (2001). Controlling the false discovery rate in behavior genetics research. Behav. Brain Res. 125, 279–284. 10.1016/s0166-4328(01)00297-2 11682119

[B11] Berens P. (2009). CircStat: a MATLAB toolbox for circular statistics. J. Stat. Softw. 31:10 10.18637/jss.v031.i10

[B12] Bokil H. Andrews P. Kulkarni J. E. Mehta S. Mitra P. P. (2010). Chronux: a platform for analyzing neural signals. J. Neurosci. Methods 192, 146–151. 10.1016/j.jneumeth.2010.06.020 20637804 PMC2934871

[B13] Bokil H. Purpura K. Schoffelen J. M. Thomson D. Mitra P. (2007). Comparing spectra and coherences for groups of unequal size. J. Neurosci. Methods 159, 337–345. 10.1016/j.jneumeth.2006.07.011 16945422

[B14] Bonnefond M. Kastner S. Jensen O. (2017). Communication between brain areas based on nested oscillations. eNeuro 4:ENEURO.0153-16.2017. 10.1523/ENEURO.0153-16.2017 28374013 PMC5367085

[B15] Brazhnik E. Novikov N. McCoy A. J. Cruz A. V. Walters J. R. (2014). Functional correlates of exaggerated oscillatory activity in basal ganglia output in hemiparkinsonian rats. Exp. Neurol. 261, 563–577. 10.1016/j.expneurol.2014.07.010 25084518 PMC4318574

[B16] Brittain J.-S. S. Brown P. (2014). Oscillations and the basal ganglia: motor control and beyond. Neuroimage 85, 637–647. 10.1016/j.neuroimage.2013.05.084 23711535 PMC4813758

[B17] Brittain J.-S. S. Sharott A. Brown P. (2014). The highs and lows of β activity in cortico-basal ganglia loops. Eur. J. Neurosci. 39, 1951–1959. 10.1111/ejn.12574 24890470 PMC4285950

[B18] Bronte-Stewart H. Barberini C. Koop M. M. Hill B. C. Henderson J. M. Wingeier B. (2009). The STN β-band profile in Parkinson’s disease is stationary and shows prolonged attenuation after deep brain stimulation. Exp. Neurol. 215, 20–28. 10.1016/j.expneurol.2008.09.008 18929561

[B19] Buzsáki G. (2004). Large-scale recording of neuronal ensembles. Nat. Neurosci. 7, 446–451. 10.1038/nn1233 15114356

[B20] Buzsáki G. Watson B. O. (2012). Brain rhythms and neural syntax: implications for efficient coding of cognitive content and neuropsychiatric disease. Dialogues Clin. Neurosci. 14, 345–367. Available online at: https://www.dialogues-cns.org/contents-14-4/dialoguesclinneurosci-14-345 23393413 10.31887/DCNS.2012.14.4/gbuzsaki PMC3553572

[B27] Cardoso de Oliveira S. Gribova A. Donchin O. Bergman H. Vaadia E. (2001). Neural interactions between motor cortical hemispheres during bimanual and unimanual arm movements. Eur. J. Neurosci. 14, 1881–1896. 10.1046/j.0953-816x.2001.01801.x 11860483

[B21] Chatrian G. E. Petersen M. C. Lazarte J. A. (1959). The blocking of the rolandic wicket rhythm and some central changes related to movement. Electroencephalogr. Clin. Neurophysiol. 11, 497–510. 10.1016/0013-4694(59)90048-3 13663823

[B22] Cincotta M. Giovannelli F. Borgheresi A. Balestrieri F. Vanni P. Ragazzoni A.. (2006). Surface electromyography shows increased mirroring in Parkinson’s disease patients without overt mirror movements. Mov. Disord. 21, 1461–1465. 10.1002/mds.20972 16705686

[B23] Crone N. E. Miglioretti D. L. Gordon B. Sieracki J. M. Wilson M. T. Uematsu S.. (1998). Functional mapping of human sensorimotor cortex with electrocorticographic spectral analysis. I. α and β event-related desynchronization. Brain 121, 2271–2299. 10.1093/brain/121.12.2271 9874480

[B24] Darvas F. Hebb A. O. (2014). Task specific inter-hemispheric coupling in human subthalamic nuclei. Front. Hum. Neurosci. 8:701. 10.3389/fnhum.2014.00701 25249965 PMC4157552

[B25] de Hemptinne C. Ryapolova-Webb E. S. Air E. L. Garcia P. A. Miller K. J. Ojemann J. G.. (2013). Exaggerated phase-amplitude coupling in the primary motor cortex in Parkinson disease. Proc. Natl. Acad. Sci. U S A 110, 4780–4785. 10.1073/pnas.1214546110 23471992 PMC3606991

[B26] de Hemptinne C. Swann N. C. Ostrem J. L. Ryapolova-Webb E. S. Luciano S. (2015). Therapeutic deep brain stimulation reduces cortical phase-amplitude coupling in Parkinson’s disease. Nat. Neurosci. 18, 779–786. 10.1038/nn.3997 25867121 PMC4414895

[B28] de Solages C. Hill B. C. Koop M. M. Henderson J. M. Bronte-Stewart H. (2010). Bilateral symmetry and coherence of subthalamic nuclei β band activity in Parkinson’s disease. Exp. Neurol. 221, 260–266. 10.1016/j.expneurol.2009.11.012 19944098

[B29] Delaville C. Cruz A. V. McCoy A. J. Brazhnik E. Avila I. Novikov N.. (2014). Oscillatory activity in basal ganglia and motor cortex in an awake behaving rodent model of Parkinson’s disease. Basal Ganglia 3, 221–227. 10.1016/j.baga.2013.12.001 25667820 PMC4319371

[B30] Desikan R. S. Ségonne F. Fischl B. Quinn B. T. Dickerson B. C. Blacker D.. (2006). An automated labeling system for subdividing the human cerebral cortex on MRI scans into gyral based regions of interest. Neuroimage 31, 968–980. 10.1016/j.neuroimage.2006.01.021 16530430

[B31] Deuschl G. Schade-Brittinger C. Krack P. Volkmann J. Schäfer H. Bötzel K.. (2006). A randomized trial of deep-brain stimulation for Parkinson’s disease. N. Engl. J. Med. 355, 896–908. 10.1056/NEJMoa060281 16943402

[B32] Devergnas A. Pittard D. Bliwise D. Wichmann T. (2014). Relationship between oscillatory activity in the cortico-basal ganglia network and parkinsonism in MPTP-treated monkeys. Neurobiol. Dis. 68, 156–166. 10.1016/j.nbd.2014.04.004 24768805 PMC4275129

[B33] Devos D. Szurhaj W. Reyns N. Labyt E. Houdayer E. Bourriez J. L.. (2006). Predominance of the contralateral movement-related activity in the subthalamo-cortical loop. Clin. Neurophysiol. 117, 2315–2327. 10.1016/j.clinph.2006.06.719 16926112

[B34] Dorval A. D. Grill W. M. (2014). Deep brain stimulation of the subthalamic nucleus reestablishes neuronal information transmission in the 6-OHDA rat model of parkinsonism. J. Neurophysiol. 111, 1949–1959. 10.1152/jn.00713.2013 24554786 PMC4044335

[B35] Duque J. Mazzocchio R. Dambrosia J. Murase N. Olivier E. Cohen L. G. (2005). Kinematically specific interhemispheric inhibition operating in the process of generation of a voluntary movement. Cereb. Cortex 15, 588–593. 10.1093/cercor/bhh160 15342437

[B36] Filion M. Tremblay L. (1991). Abnormal spontaneous activity of globus pallidus neurons in monkeys with MPTP-induced parkinsonism. Brain Res. 547, 142–151. 10.1016/0006-8993(91)90585-j 1677607

[B37] Fries P. (2005). A mechanism for cognitive dynamics: neuronal communication through neuronal coherence. Trends Cogn. Sci. 9, 474–480. 10.1016/j.tics.2005.08.011 16150631

[B38] Gastaut H. (1952). Electrocorticographic study of the reactivity of rolandic rhythm. Rev. Neurol. Paris. 87, 176–192. 13014777

[B39] Genovese C. R. Lazar N. A. Nichols T. (2002). Thresholding of statistical maps in functional neuroimaging using the false discovery rate. Neuroimage 15, 870–878. 10.1006/nimg.2001.1037 11906227

[B40] Goldfine A. M. Victor J. D. Conte M. M. Bardin J. C. Schiff N. D. (2011). Determination of awareness in patients with severe brain injury using EEG power spectral analysis. Clin. Neurophysiol. 122, 2157–2168. 10.1016/j.clinph.2011.03.022 21514214 PMC3162107

[B41] Gross J. Pollok B. Dirks M. Timmermann L. Butz M. Schnitzler A. (2005). Task-dependent oscillations during unimanual and bimanual movements in the human primary motor cortex and SMA studied with magnetoencephalography. Neuroimage 26, 91–98. 10.1016/j.neuroimage.2005.01.025 15862209

[B42] Hebb A. O. Darvas F. Miller K. J. (2012). Transient and state modulation of β power in human subthalamic nucleus during speech production and finger movement. Neuroscience 202, 218–233. 10.1016/j.neuroscience.2011.11.072 22173017 PMC3286522

[B43] Holm S. (1979). A simple sequential rejective multiple test procedure. Scand. J. Stat. 6, 65–70.

[B44] Hoover J. E. Strick P. L. (1993). Multiple output channels in the basal ganglia. Science 259, 819–821. 10.1126/science.7679223 7679223

[B45] Jensen O. Colgin L. L. (2007). Cross-frequency coupling between neuronal oscillations. Trends Cogn. Sci. 11, 267–269. 10.1016/j.tics.2007.05.003 17548233

[B46] Johnson K. A. Cunnington R. Bradshaw J. L. Phillips J. G. Iansek R. Rogers M. A. (1998). Bimanual co-ordination in Parkinson’s disease. Brain 121, 743–753. 10.1093/brain/121.4.743 9577398

[B47] Kato K. Yokochi F. Iwamuro H. Kawasaki T. Hamada K. Isoo A.. (2016). Frequency-specific synchronization in the bilateral subthalamic nuclei depending on voluntary muscle contraction and relaxation in patients with Parkinson’s disease. Front. Hum. Neurosci. 10:131. 10.3389/fnhum.2016.00131 27064969 PMC4811912

[B48] Kato K. Yokochi F. Taniguchi M. Okiyama R. Kawasaki T. Kimura K.. (2015). Bilateral coherence between motor cortices and subthalamic nuclei in patients with Parkinson’s disease. Clin. Neurophysiol. 126, 1941–1950. 10.1016/j.clinph.2014.12.007 25591829

[B49] Kondylis E. D. Randazzo M. J. Alhourani A. Lipski W. J. Wozny T. A. Pandya Y.. (2016). Movement-related dynamics of cortical oscillations in Parkinson’s disease and essential tremor. Brain 139, 2211–2223. 10.1093/brain/aww144 27329771 PMC5022700

[B50] Kraft E. Chen A. W. Flaherty A. W. Blood A. J. Kwong K. K. Jenkins B. G. (2007). The role of the basal ganglia in bimanual coordination. Brain Res. 1151, 62–73. 10.1016/j.brainres.2007.01.142 17448452

[B51] Lalo E. Thobois S. Sharott A. Polo G. Mertens P. Pogosyan A.. (2008). Patterns of bidirectional communication between cortex and basal ganglia during movement in patients with Parkinson disease. J. Neurosci. 28, 3008–3016. 10.1523/JNEUROSCI.5295-07.2008 18354004 PMC6670699

[B52] Lehéricy S. Bardinet E. Tremblay L. Van de Moortele P. F. Pochon J. B. Dormont D.. (2006). Motor control in basal ganglia circuits using fMRI and brain atlas approaches. Cereb. Cortex 16, 149–161. 10.1093/cercor/bhi089 15858164

[B53] Leocani L. Cohen L. G. Wassermann E. M. Brain I.-K. (2000). Human corticospinal excitability evaluated with transcranial magnetic stimulation during different reaction time paradigms. Brain 123, 1161–1173. 10.1093/brain/123.6.1161 10825355

[B54] Liepert J. Dettmers C. Terborg C. Weiller C. (2001). Inhibition of ipsilateral motor cortex during phasic generation of low force. Clin. Neurophysiol. 112, 114–121. 10.1016/s1388-2457(00)00503-4 11137668

[B55] Little S. Brown P. (2014). The functional role of β oscillations in Parkinson’s disease. Parkinsonism Relat. Disord. 20, S44–S48. 10.1016/s1353-8020(13)70013-0 24262186

[B56] Little S. Tan H. Anzak A. Pogosyan A. Kühn A. Brown P. (2013). Bilateral functional connectivity of the basal ganglia in patients with Parkinson’s disease and its modulation by dopaminergic treatment. PLoS One 8:e82762. 10.1371/journal.pone.0082762 24376574 PMC3869733

[B57] López-Azcárate J. Tainta M. Rodríguez-Oroz M. C. Valencia M. González R. Guridi J.. (2010). Coupling between β and high-frequency activity in the human subthalamic nucleus may be a pathophysiological mechanism in Parkinson’s disease. J. Neurosci. 30, 6667–6677. 10.1523/JNEUROSCI.5459-09.2010 20463229 PMC6632566

[B58] Malekmohammadi M. AuYong N. Price C. M. Tsolaki E. Hudson A. E. Pouratian N. (2018a). Propofol-induced changes in α-β sensorimotor cortical connectivity. Anesthesiology 128, 305–316. 10.1097/ALN.0000000000001940 29068830 PMC5771969

[B59] Malekmohammadi M. AuYong N. Ricks-Oddie J. Bordelon Y. Pouratian N. (2018b). Pallidal deep brain stimulation modulates excessive cortical high β phase amplitude coupling in Parkinson disease. Brain Stimul. 11, 607–617. 10.1016/j.brs.2018.01.028 29422442 PMC5930048

[B60] Malekmohammadi M. Elias W. J. Pouratian N. (2015). Human thalamus regulates cortical activity via spatially specific and structurally constrained phase-amplitude coupling. Cereb. Cortex 25, 1618–1628. 10.1093/cercor/bht358 24408958 PMC4447821

[B61] Maris E. Fries P. Van Ede F. (2016). Diverse phase relations among neuronal rhythms and their potential function. Trends Neurosci. 39, 86–99. 10.1016/j.tins.2015.12.004 26778721

[B62] Moran A. Stein E. Tischler H. Bar-Gad I. (2012). Decoupling neuronal oscillations during subthalamic nucleus stimulation in the parkinsonian primate. Neurobiol. Dis. 45, 583–590. 10.1016/j.nbd.2011.09.016 22001603

[B63] Muakkassa K. F. Strick P. L. (1979). Frontal lobe inputs to primate motor cortex: evidence for four somatotopically organized ‘premotor’ areas. Brain Res. 177, 176–182. 10.1016/0006-8993(79)90928-4 115545

[B64] Murthy V. N. Fetz E. E. (1996). Synchronization of neurons during local field potential oscillations in sensorimotor cortex of awake monkeys. J. Neurophysiol. 76, 3968–3982. 10.1152/jn.1996.76.6.3968 8985893

[B65] Nambu A. Tokuno H. Takada M. (2002). Functional significance of the cortico-subthalamo-pallidal ‘hyperdirect’ pathway. Neurosci. Res. 43, 111–117. 10.1016/s0168-0102(02)00027-5 12067746

[B66] Neumann W. J. Jha A. Bock A. Huebl J. Horn A. Schneider G. H.. (2015). Cortico-pallidal oscillatory connectivity in patients with dystonia. Brain 138, 1894–1906. 10.1093/brain/awv109 25935723

[B67] Niedermeyer E. (1997). α rhythms as physiological and abnormal phenomena. Int. J. Psychophysiol. 26, 31–49. 10.1016/s0167-8760(97)00754-x 9202993

[B68] Nini A. Feingold A. Slovin H. Bergman H. (1995). Neurons in the globus pallidus do not show correlated activity in the normal monkey, but phase-locked oscillations appear in the MPTP model of parkinsonism. J. Neurophysiol. 74, 1800–1805. 10.1152/jn.1995.74.4.1800 8989416

[B69] Nolte G. Bai O. Wheaton L. Mari Z. Vorbach S. Hallett M. (2004). Identifying true brain interaction from EEG data using the imaginary part of coherency. Clin. Neurophysiol. 115, 2292–2307. 10.1016/j.clinph.2004.04.029 15351371

[B70] Oostenveld R. Fries P. Maris E. Schoffelen J. M. (2011). FieldTrip: open source software for advanced analysis of MEG, EEG, and invasive electrophysiological data. Comput. Intell. Neurosci. 2011:156869. 10.1155/2011/156869 21253357 PMC3021840

[B71] Palmer S. J. Eigenraam L. Hoque T. McCaig R. G. Troiano A. McKeown M. J. (2009). Levodopa-sensitive, dynamic changes in effective connectivity during simultaneous movements in Parkinson’s disease. Neuroscience 158, 693–704. 10.1016/j.neuroscience.2008.06.053 18722512

[B72] Parent A. Hazrati L. N. (1995). Functional-anatomy of the basal ganglia. 1. The cortico-basal ganglia-thalamo-cortical loop. Brain Res. Rev. 20, 91–127. 10.1016/0165-0173(94)00007-c 7711769

[B73] Pollok B. Krause V. Martsch W. Wach C. Schnitzler A. Südmeyer M. (2012). Motor-cortical oscillations in early stages of Parkinson’s disease. J. Physiol. 590, 3203–3212. 10.1113/jphysiol.2012.231316 22547636 PMC3406400

[B74] Pollok B. Südmeyer M. Gross J. Schnitzler A. (2005). The oscillatory network of simple repetitive bimanual movements. Cogn. Brain Res. 25, 300–311. 10.1016/j.cogbrainres.2005.06.004 16023333

[B75] Sanes J. N. Donoghue J. P. (1993). Oscillations in local field potentials of the primate motor cortex during voluntary movement. Proc. Natl. Acad. Sci. U S A 90, 4470–4474. 10.1073/pnas.90.10.4470 8506287 PMC46533

[B76] Schnitzler A. Gross J. (2005). Normal and pathological oscillatory communication in the brain. Nat. Rev. Neurosci. 6, 285–296. 10.1038/nrn1650 15803160

[B77] Scholz V. H. Flaherty A. W. Kraft E. Keltner J. R. Kwong K. K. Chen Y. I.. (2000). Laterality, somatotopy and reproducibility of the basal ganglia and motor cortex during motor tasks. Brain Res. 879, 204–215. 10.1016/s0006-8993(00)02749-9 11011024

[B78] Sharott A. Magill P. J. Bolam P. J. Brown P. (2005). Directional analysis of coherent oscillatory field potentials in the cerebral cortex and basal ganglia of the rat. J. Physiol. 562, 951–963. 10.1113/jphysiol.2004.073189 15550466 PMC1665537

[B79] Shibasaki H. Hallett M. (2006). What is the bereitschaftspotential? Clin. Neurophysiol. 117, 2341–2356. 10.1016/j.clinph.2006.04.025 16876476

[B80] Stein E. Bar-Gad I. (2013). β oscillations in the cortico-basal ganglia loop during parkinsonism. Exp. Neurol. 245, 52–59. 10.1016/j.expneurol.2012.07.023 22921537

[B81] Swann N. C. de Hemptinne C. Aron A. R. Ostrem J. L. Knight R. T. Starr P. A. (2015). Elevated synchrony in Parkinson disease detected with electroencephalography. Ann. Neurol. 78, 742–750. 10.1002/ana.24507 26290353 PMC4623949

[B82] Tiesinga P. H. Sejnowski T. J. (2010). Mechanisms for phase shifting in cortical networks and their role in communication through coherence. Front. Hum. Neurosci. 4:196. 10.3389/fnhum.2010.00196 21103013 PMC2987601

[B83] Tort A. B. L. Komorowski R. Eichenbaum H. Kopell N. (2010). Measuring phase-amplitude coupling between neuronal oscillations of different frequencies. J. Neurophysiol. 104, 1195–1210. 10.1152/jn.00106.2010 20463205 PMC2941206

[B84] Tremblay L. O. Filion M. BéDard P. J. (1989). Responses of pallidal neurons to striatal stimulation in monkeys with MPTP-induced parkinsonism. Brain Res. 498, 17–33. 10.1016/0006-8993(89)90395-8 2790469

[B85] Tsiokos C. Hu X. Pouratian N. (2013). 200–300Hz movement modulated oscillations in the internal globus pallidus of patients with Parkinson’s disease. Neurobiol. Dis. 54, 464–474. 10.1016/j.nbd.2013.01.020 23388190 PMC3629001

[B86] Tsiokos C. Malekmohammadi M. AuYong N. Pouratian N. (2017). Pallidal low β-low γ phase-amplitude coupling inversely correlates with Parkinson disease symptoms. Clin. Neurophysiol. 128, 2165–2178. 10.1016/j.clinph.2017.08.001 28942154 PMC5675765

[B87] van Wijk B. C. M. Beudel M. Jha A. Oswal A. Foltynie T. Hariz M. I.. (2016). Subthalamic nucleus phase-amplitude coupling correlates with motor impairment in Parkinson’s disease. Clin. Neurophysiol. 127, 2010–2019. 10.1016/j.clinph.2016.01.015 26971483 PMC4803022

[B88] van Wijk B. C. M. Neumann W.-J. Schneider G.-H. Sander T. H. Litvak V. Kühn A. A. (2017). Low-β cortico-pallidal coherence decreases during movement and correlates with overall reaction time. Neuroimage 159, 1–8. 10.1016/j.neuroimage.2017.07.024 28712991 PMC5678295

[B89] von Nicolai C. Engler G. Sharott A. Engel A. K. Moll C. K. Siegel M. (2014). Corticostriatal coordination through coherent phase-amplitude coupling. J. Neurosci. 34, 5938–5948. 10.1523/JNEUROSCI.5007-13.2014 24760853 PMC6608287

[B90] Wannier T. Liu J. Morel A. Jouffrais C. Rouiller E. M. (2002). Neuronal activity in primate striatum and pallidum related to bimanual motor actions. Neuroreport 13, 143–147. 10.1097/00001756-200201210-00033 11924876

[B91] Weaver F. M. Follett K. Stern M. Hur K. Harris C. Marks W. J.. (2009). Bilateral deep brain stimulation vs. best medical therapy for patients with advanced Parkinson disease: a randomized controlled trial. JAMA 301, 63–73. 10.1001/jama.2008.929 19126811 PMC2814800

[B92] Wichmann T. Soares J. (2006). Neuronal firing before and after burst discharges in the monkey basal ganglia is predictably patterned in the normal state and altered in parkinsonism. J. Neurophysiol. 95, 2120–2133. 10.1152/jn.01013.2005 16371459

[B93] Williams D. Kuhn A. Kupsch A. Tijssen M. van Bruggen G. Speelman H.. (2005). The relationship between oscillatory activity and motor reaction time in the parkinsonian subthalamic nucleus. Eur. J. Neurosci. 21, 249–258. 10.1111/j.1460-9568.2004.03817.x 15654862

[B94] Womelsdorf T. Schoffelen J. M. Oostenveld R. Singer W. Desimone R. Engel A. K.. (2007). Modulation of neuronal interactions through neuronal synchronization. Science 316, 1609–1612. 10.1126/science.1139597 17569862

[B95] Wu T. Hou Y. Hallett M. Zhang J. Chan P. (2015). Lateralization of brain activity pattern during unilateral movement in Parkinson’s disease. Hum. Brain Mapp. 36, 1878–1891. 10.1002/hbm.22743 25644527 PMC6869082

[B96] Yang A. I. Vanegas N. Lungu C. Zaghloul K. A. (2014). β-coupled high-frequency activity and β-locked neuronal spiking in the subthalamic nucleus of Parkinson’s disease. J. Neurosci. 34, 12816–12827. 10.1523/JNEUROSCI.1895-14.2014 25232117 PMC4166162

